# Enantiospecific pharmacokinetics and drug–drug interactions of primaquine and blood-stage antimalarial drugs

**DOI:** 10.1093/jac/dky297

**Published:** 2018-08-03

**Authors:** Kalayanee Chairat, Podjanee Jittamala, Borimas Hanboonkunupakarn, Sasithon Pukrittayakamee, Warunee Hanpithakpong, Daniel Blessborn, Nicholas J White, Nicholas P J Day, Joel Tarning

**Affiliations:** 1 Mahidol-Oxford Tropical Medicine Research Unit, Faculty of Tropical Medicine, Mahidol University, Bangkok, Thailand; 2 Department of Tropical Hygiene, Faculty of Tropical Medicine, Mahidol University, Bangkok, Thailand; 3 Department of Clinical Tropical Medicine, Faculty of Tropical Medicine, Mahidol University, Bangkok, Thailand; 4 Centre for Tropical Medicine and Global Health, Nuffield Department of Medicine, University of Oxford, Oxford, UK

## Abstract

**Objectives:**

Characterization of the pharmacokinetic properties of the enantiomers of primaquine and carboxyprimaquine following administration of racemic primaquine given alone and in combination with commonly used antimalarial drugs.

**Methods:**

Enantiomeric pharmacokinetics were evaluated in 49 healthy adult volunteers enrolled in three randomized cross-over studies in which a single dose of primaquine was given alone and then, after a suitable washout period, in combination with chloroquine, dihydroartemisinin/piperaquine or pyronaridine/artesunate. Non-linear mixed-effects modelling was used to characterize pharmacokinetics and assess the impact of drug–drug interactions.

**Results:**

The volume of distribution of racemic primaquine was decreased by a median (95% CI) of 22.0% (2.24%–39.9%), 24.0% (15.0%–31.5%) and 25.7% (20.3%–31.1%) when co-administered with chloroquine, dihydroartemisinin/piperaquine and pyronaridine/artesunate, respectively. The oral clearance of primaquine was decreased by a median of 19.1% (14.5%–22.8%) when co-administered with pyronaridine/artesunate. These interactions were enantiospecific with a relatively higher effect on (+)-*S*-primaquine than on (−)-*R*-primaquine. No drug–drug interaction effects were seen on the pharmacokinetics of either carboxyprimaquine enantiomer.

**Conclusions:**

Population pharmacokinetic models characterizing the enantiospecific properties of primaquine were developed successfully. Exposure to primaquine, particularly to the (+)-*S*-primaquine but not the carboxy metabolites, increased by up to 30% when co-administered with commonly used antimalarial drugs. A better mechanistic understanding of primaquine metabolism is required for assessment of its efficacy and haematological toxicity in humans.

## Introduction

The 8-aminoquinoline primaquine is the only approved drug active against the dormant liver-stage malaria parasites (i.e. hypnozoites) of *Plasmodium vivax* and *Plasmodium ovale*. Primaquine also has significant asexual-stage activity against these parasites. It is used for the radical treatment of *P. vivax* and *P. ovale* malaria together with blood-stage schizontocidal drugs such as chloroquine or artemisinin-based combination therapies (ACTs). The WHO recommends 0.25–0.5 mg of primaquine base/kg body weight once daily for 14 days for the treatment of *P. vivax* and *P. ovale* malaria in children and adults. Primaquine causes dose-dependent haemolysis in people with glucose-6-phosphate dehydrogenase (G6PD) deficiency. This enzymopathy is common in tropical areas, with prevalences between 3% and 30%. To mitigate the haemolytic risk in individuals with milder variants of G6PD deficiency, once-weekly primaquine (0.75 mg base/kg) for 8 weeks is recommended.^1^ Primaquine is also an effective gametocytocidal drug, eliminating mature *Plasmodium falciparum* gametocytes, and thereby reducing transmission of malaria from humans to mosquitoes. A single low dose of primaquine (0.25 mg base/kg) is considered to be as effective as the previously recommended dose of 0.75 mg base/kg in blocking transmission, with a very low risk of haemolysis in G6PD-deficient individuals.^2–4^ Recent studies confirm that the addition of a single low dose of primaquine (0.25 mg base/kg) to artemether/lumefantrine and dihydroartemisinin/piperaquine treatments in *P. falciparum* malaria reduced gametocytaemia and transmission of malaria to mosquitoes compared with the ACT treatment alone.^5^^,^^6^

Primaquine phosphate formulations used clinically are racemic (50:50) mixtures of (+)-*S*- and (−)-*R*-enantiomers. The drug is almost completely absorbed after oral administration (96% bioavailability)^7^ and is readily metabolized in the liver. The major metabolite, carboxyprimaquine, is formed principally by hepatic monoamine oxidase A (MAO-A).^8^^,^^9^ Carboxyprimaquine is believed to be pharmacologically inactive and non-toxic.^10^^,^^11^ In contrast, reactive ring-hydroxylated primaquine metabolites, generated through cytochrome P450 (CYP) (mainly 2D6)-mediated metabolism, are thought to mediate the anti-hypnozoite and gametocytocidal activities and also the haemolytic toxicity in G6PD-deficient individuals.^12–16^

There is evidence to suggest that the metabolism of primaquine is enantioselective. *In vitro* metabolism studies using rat liver fractions and human hepatocytes showed that (−)-*R*-primaquine was preferentially converted into (−)-*R*-carboxyprimaquine.^17–19^ Studies of CYP2D6-mediated metabolism *in vitro* revealed that (+)-*S*-primaquine preferentially generated 2- and 5-hydroxyprimaquine while (−)-*R*-primaquine preferentially generated 3- and 4-hydroxyprimaquine.^20^ However, the relative contributions of each of the hydroxylated primaquine metabolites to antimalarial activity and haemolytic toxicity are still unclear. The first evidence of different pharmacokinetic profiles of primaquine enantiomers in humans (*n *=* *6) after racemic primaquine administration showed higher exposure to (+)-*S*-primaquine than to (−)-*R*-primaquine, and only (−)-*R*-carboxyprimaquine was detected in plasma.^21^

Despite its extensive use as a partner drug with other antimal-arials, there are few studies of primaquine drug–drug interactions. Non-compartmental pharmacokinetic analyses assessing interactions between primaquine and chloroquine, dihydroartemisinin/piperaquine (Eurartesim^®^) and pyronaridine/artesunate (Pyramax^®^) all suggested higher primaquine exposures when administered in combination compared with when primaquine was administered alone.^22–24^ Enantiospecific pharmacokinetic interactions were not addressed in these studies. The objective of this study was to characterize the pharmacokinetic properties of racemic and enantiomeric primaquine and carboxyprimaquine, following administration of racemic primaquine, and to investigate interactions with the three blood-stage antimalarial drug combinations using non-linear mixed-effects population pharmacokinetic modelling.

## Methods

### Study design and participants

Data from three individual studies investigating potential pharmacokinetic drug–drug interactions of racemic primaquine when co-administered with chloroquine (Study I, Clinicaltrials.gov identifier NCT01218932), dihydroartemisinin/piperaquine (Study II, Clinicaltrials.gov identifier NCT01525511) and pyronaridine/artesunate (Study III, Clinicaltrials.gov identifier NCT01552330) were pooled to develop population pharmacokinetic models of primaquine and carboxyprimaquine, and their enantiomers. These studies were randomized, open-label, two-arm, three-period, single-dose cross-over studies in healthy G6PD-normal Thai adult volunteers conducted at the Clinical Therapeutics Unit, Mahidol-Oxford Tropical Medicine Research Unit, Bangkok, Thailand. Complete details of study design, inclusion criteria, and non-compartmental analysis results have been reported in full elsewhere.^22–24^

In each study, 16–17 volunteers were randomized to receive primaquine alone, primaquine combined with co-administered drug, and co-administered drug alone. The dose of each drug was as follows: 30 mg of primaquine; 600 mg of chloroquine; 120 mg of dihydroartemisinin plus 960 mg of piperaquine tetraphosphate; and 540 mg of pyronaridine tetraphosphate plus 180 mg of artesunate. Washout was 7 days after primaquine alone and 8 weeks after chloroquine-, piperaquine- and pyronaridine-containing regimens. The study drugs were administered as a single oral dose 30 min after a light standard meal followed by 4 h of fasting after dosing.

### Ethics

All studies were conducted in accordance with good clinical practice and the guiding principles of the Declaration of Helsinki and were approved by the Ethics Committee of the Faculty of Tropical Medicine, Mahidol University (Study I, MUTM 2010-028-01; Study II, MUTM 2012-009-01; Study III, MUTM 2012-013-01) and the Oxford Tropical Research Ethics Committee, University of Oxford (Study I, OXTREC 39-10; Study II, OXTREC 58-11; Study III, OXTREC 04-12). Signed and dated written informed consent was obtained from each participant prior to participation in the study.

### Drug analysis

Blood samples for plasma drug concentration measurements were collected pre-dose and at 0.25, 0.5, 1, 1.5, 2, 3, 4, 6, 8, 10, 12 and 24 h and on day 3 (48–54 h) post-dose. Additional blood samples were collected on days 4, 7, 11, 15, 22, 36 and 42 when primaquine was combined with co-administered drugs. The pharmacokinetic properties of the co-administered antimalarial drugs have been reported previously.^22–25^

Determination of racemic and enantiomeric primaquine and carboxyprimaquine concentrations in plasma samples was performed using an LC–MS/MS method that was validated according to US FDA guidelines (W. Hanpithakpong, N. P. Day, N. J. White and J. Tarning, unpublished data). Racemic primaquine and carboxyprimaquine were provided by the Worldwide Antimalarial Resistance Network (WWARN). Enantiomeric primaquine and carboxyprimaquine and their stable isotope-labelled internal standards (6-trideuteromethoxyprimaquine diphosphate and 6-trideuteromethoxycarboxyprimaquine) were obtained from the National Centre for Natural Products Research (University of Mississippi, MS, USA). Plasma samples were processed by protein precipitation followed by phospholipid removal by solid-phase extraction. Racemic separations were performed using a Hypersil GOLD column [100 mm × 4.6 mm, internal diameter (ID) 3 μm; Thermo Scientific, Fremont, CA, USA] with 10 mM ammonium acetate/acetonitrile (50:50 v/v) at a flow rate of 0.5 μL/min in isocratic elution mode for 8 min. Enantiomeric separations were achieved using a CHIRALCEL OD-3R column (150 mm × 2.1 mm, ID 3 μm; Chiral Technologies Inc., PA, USA) with 20 mM ammonium formate with 0.1% acetic acid/acetonitrile (75:25 v/v) and methanol/acetonitrile (75:25 v/v) at a flow rate of 1.0 mL/min in gradient elution mode for 26 min.

Detection was performed using an API 5000 triple-quadrupole system (Applied Biosystems/MDS Sciex, Foster City, CA, USA) operated in the positive electrospray ionization mode. Quantification was performed using multiple reaction monitoring of the transitions at *m*/*z* 260–175 and 263–86 for primaquine and its internal standard, respectively, and at *m*/*z* 275–175 and 278–178 for carboxyprimaquine and its internal standard, respectively. The within-day and between-day precisions of racemic and enantiomeric primaquine and carboxyprimaquine were <9% at all tested quality control concentrations and <15% at the lower limit of quantification (LLOQ). The LLOQ was 1.14 and 4.88 ng/mL for racemic primaquine and carboxyprimaquine, respectively, and 0.571 and 2.44 ng/mL for enantiomeric primaquine and carboxyprimaquine, respectively.

### Population pharmacokinetic analysis

Concentrations of racemic and enantiomeric primaquine and carboxyprimaquine were converted into equivalent molar units and transformed to their natural logarithms before analysis. Population pharmacokinetic models were developed using non-linear mixed-effects modelling in NONMEM version 7.3 (ICON Development Solutions, Ellicott City, MD, USA).^26^ The first-order conditional estimation method with interactions (FOCE-I) and subroutine ADVAN5 TRANS1 were used throughout the study. Data post-processing was performed using Perl-speaks-NONMEM (PsN)^27^^,^^28^ version 4.6.0 and Xpose^29^ version 4.0 in the programming language R version 3.3.0.^30^

Racemic and enantiomeric entities were modelled separately. Racemic primaquine and carboxyprimaquine concentrations were available for plasma samples from Study I, and enantiomeric concentrations were available for plasma samples from Study II and Study III. Therefore, the sum of the concentrations of each enantiomer was used in the racemic models.

A simultaneous parent–metabolite model was developed to describe the pharmacokinetics of racemic and enantiomeric primaquine and carboxyprimaquine. Complete *in vivo* conversion of primaquine to carboxyprimaquine was assumed to maintain structural identifiability. Enantiomeric primaquine and carboxyprimaquine were modelled assuming a complete conversion of (−)-*R*-primaquine to (−)-*R*-carboxyprimaquine and likewise for the (+)-*S*-enantiomers.

The structural base models were parameterized as primaquine first-order absorption rate constant (*k*_a_), relative oral bioavailability (*F*), elimination clearance of primaquine (CL/*F*_PRQ_) and carboxyprimaquine (CL/*F*_CPRQ_), and volume of distribution of primaquine (*V*/*F*_PRQ_) and carboxyprimaquine (*V*/*F*_CPRQ_). Since intravenous data were not available, *F* was fixed to unity for the population but inter-individual variability (IIV) was allowed. Different distribution models (one-, two- and three-compartment) and absorption models (zero-order, first-order absorption with and without lag time and a flexible transit compartment model^31^) were evaluated. For the transit compartment absorption model, the number of transit compartments was determined by stepwise addition. Mean absorption transit time (MTT) was estimated as the number of transit compartments divided by the transit rate constant (*k*_tr_). A model incorporating a first-pass effect was evaluated where an estimated fraction of the absorbed oral primaquine was converted into carboxyprimaquine during the first-pass hepatic metabolism (*F*_m_), while the remaining fraction of primaquine (1 − *F*_m_) was absorbed unchanged. IIV and inter-occasion variability (IOV) were implemented exponentially, and assumed to be independent and normally distributed around zero with variance *ω*2. Primaquine taken alone and when co-administered with other antimalarial drugs were regarded as separate occasions in the same individual. Residual variability was modelled as separate additive errors for each entity on log-transformed data, which essentially correspond to exponential residual errors on the normal scale. The level 2 (L2) data item in NONMEM was used to account for potential correlations between observations (i.e. residual errors) of parent drug and its metabolite when their measurements were from the same plasma samples.

The best structural base models were used subsequently for covariate model building. Body weight, centred on the median value of the population, was implemented *a priori* using allometric scaling with a fixed exponent of 0.75 on clearance parameters and 1 on volume parameters.^32^ Possible drug–drug interactions were modelled as proportional effects on each parameter. Other covariates, including age, gender and baseline haematological and serum chemistry measurements, were screened based on physiological plausibility and statistical significance (*P *<* *0.05, using Pearson’s, Spearman’s and Kendall’s parameter–covariate correlations) and evaluated formally as linear, exponential or power relationships using a stepwise covariate model with forward addition (*P *<* *0.05) and backward deletion (*P *<* *0.001). In addition, a full covariate model was constructed separately to estimate the impact of each co-administration by including the covariate simultaneously on all parameters, except *F*, in order to retain model identifiability. The 95% CI of the estimated covariate effect was obtained by bootstrapping the full model (*n *=* *1000). A significant drug–drug interaction effect was defined as a difference of at least 20% in parameter estimates. Secondary pharmacokinetic parameters, including plasma *C*_max_, plasma *T*_max_, terminal elimination *t*_1/2_ and total plasma AUC, were calculated from the individual *post hoc* empirical Bayes estimates (EBEs) of primary parameters from the final models.

The objective function value (OFV; equal to −2 log-likelihood) was used to discriminate between two competing hierarchical models. A decrease in OFV (*Δ*OFV) of 3.84, 6.63 and 10.8 was considered a significant improvement in model fit at *P *<* *0.05, *P *<* *0.01 and *P *<* *0.001, respectively, with one additional parameter. Physiological plausibility, precision of parameter estimates and goodness-of-fit (GOF) were employed to guide model selection. Shrinkage in random effects was calculated to assess the reliability of individual parameter estimates (EBEs) and GOF diagnostics.^33^ Non-parametric bootstrapping (*n *=* *1000), stratified by study, was used to calculate CIs and relative standard errors (RSEs) of parameter estimates of the final models. The predictive performance of the model was examined by using simulation-based diagnostics (numerical and visual predictive checks).^34^

## Results

A total of 49 healthy volunteers (32 females and 17 males) from three separate studies were included in this population pharmacokinetic analysis. The median (range) age was 32 years (20–51 years) and the median weight was 60.2 kg (46.8–71.4 kg). Full demographic data and non-compartmental pharmacokinetic analyses assessing drug–drug interactions between primaquine and chloroquine, dihydroartemisinin/piperaquine and pyronaridine/artesunate have been published in full elsewhere.^22–24^ There were no serious adverse events. The collected data consisted of 1274 observations for each of the racemic plasma concentrations of primaquine and carboxyprimaquine and 830 observations for each enantiomeric primaquine and carboxyprimaquine plasma concentration.

### Pharmacokinetics of racemic and enantiomeric primaquine and carboxyprimaquine

**Figure 1.**
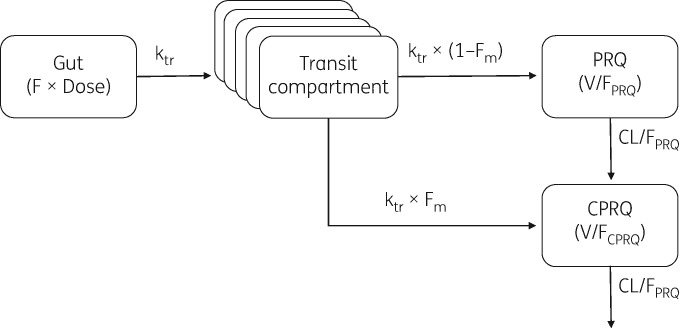
Structural representation of the final population pharmacokinetic model for racemic and enantiomeric primaquine (PRQ) and carboxyprimaquine (CPRQ).

The disposition pharmacokinetics of primaquine and carboxyprimaquine, in racemic and enantiomeric models, were both best described by a one-compartment model with first-order absorption and elimination. A two-compartment model for primaquine did not improve the model fit significantly (*P *>* *0.05). A two-compartment disposition model for carboxyprimaquine improved the model fit significantly (*Δ*OFV of −84.7) for the racemic model but *V*/*F* of the central compartment and its IIV were estimated with poor precision (RSE >100%). For enantiomeric models, the sampling periods were too short to allow precise determination of a potential peripheral compartment. The one-compartment disposition model for carboxyprimaquine showed no systematic bias in the racemic or enantiomeric models and was therefore carried forward. Oral absorption was best described by a transit-compartment model, with *k*_a_ set as equal to *k*_tr_ for model stability. The number of transit compartments was fixed to five for the racemic and (−)-*R*-enantiomer models and to six for the (+)-*S*-enantiomer model. Incorporation of hepatic first-pass metabolism showed a significant improvement (*P *<* *0.05) in model fit (*Δ*OFV of −260, −11.1 and −42.5 for racemic, (+)-*S*- and (−)-*R*-enantiomer models, respectively). A schematic representation of the final structural model is depicted in Figure 1. Only a small number of observations were below the LLOQ (<5% of total observations) and therefore excluded from the analysis. A relatively larger fraction of observations were measured to be below the LLOQ in the terminal elimination phase (28.9% of primaquine concentrations at 48–54 h after dosing, and 18.8% of carboxyprimaquine concentrations at 144 h after dosing) but the exclusion of these LLOQ data did not show any systemic bias in simulation-based diagnostics [i.e. categorical visual predictive checks (VPCs), data not shown]. Adding IOV in MTT between occasions when primaquine was taken alone and when it was co-administered with other antimalarial drugs improved the model fit significantly (*Δ*OFV of −1330, −973 and −1070 for racemic, (+)-*S*- and (−)-*R*-enantiomer models, respectively). Additional IOV in *F*_m_ also resulted in significantly improved models (ΔOFV of −295, −155 and −45.8 for racemic, (+)-*S*- and (−)-*R*-enantiomer models, respectively). Addition of correlation between parent and metabolite residual errors (i.e. L2 data item) improved the model fit significantly (*Δ*OFV of 1160, 83.2 and 266 for racemic, (+)-*S*- and (−)-*R*-enantiomer models, respectively).

The mean *V*/*F*_PRQ_ of (+)-*S*-primaquine (126 L) was lower than that of (−)-*R*-primaquine (160 L). The mean CL/*F*_PRQ_ of (+)-*S*-primaquine (12.5 L/h) was ∼2-fold lower than that of (−)-*R*-primaquine (23.1 L/h). The metabolite/parent AUC ratio (AUC_CPRQ_/AUC_PRQ_) was 35.2, 1.98 and 109 for racemic, (+)-*S*- and (−)-*R*-primaquine, respectively. 

Body weight was implemented *a priori* as an allometric function on volume and clearance parameters and resulted in an improvement in the population models (*Δ*OFV of −12.9, −4.92 and −21.1 for racemic, (+)-*S*- and (−)-*R*-enantiomer models, respectively). Using a stepwise covariate modelling approach, we found that co-administration of all three commonly used antimalarial treatments decreased *V*/*F*_PRQ_ significantly, and that co-administration of primaquine with pyronaridine/artesunate significantly decreased CL/*F*_PRQ_. Furthermore, the drug–drug interactions did not affect (+)-*S*- and (−)-*R*-primaquine equally (Table 1). No other covariates had significant effects on model parameters.

**Table 1.** Population pharmacokinetic parameter estimates from the final population pharmacokinetic models

	Racemic		(+)-*S*-enantiomer		(**−**)-*R*-enantiomer	
Parameter	estimate^a^ (%RSE^b^)	95% CI^b^	estimate^a^ (%RSE^b^)	95% CI^b^	estimate^a^ (%RSE^b^)	95% CI^b^
Fixed effects						
*F* (%)	*100 fixed*	NA	*100 fixed*	NA	*100 fixed*	NA
MTT (h)	0.893 (5.47)	0.796–0.986	0.812 (5.31)	0.734–0.901	0.833 (5.61)	0.743–0.927
No. of transit compartments	*5 fixed*	NA	*6 fixed*	NA	*5 fixed*	NA
CL/*F*_PRQ_ (L/h)	16.0 (5.31)	14.4–17.8	12.5 (5.58)	11.2–14.0	23.1 (5.94)	20.7 –26.1
*V*/*F*_PRQ_ (L)	156 (5.64)	139–173	129 (5.48)	117–145	160 (7.27)	139–184
*F*_m_ (%)	0.360 (4.83)	0.327–0.394	0.298 (6.77)	0.255–0.335	0.460 (5.08)	0.416–0.506
CL/*F*_CPRQ_ (L/h)	0.729 (3.49)	0.680–0.780	8.11 (7.14)	6.96–9.27	0.377 (4.62)	0.343–0.411
*V*/*F*_CPRQ_ (L)	16.5 (2.70)	15.7–17.5	159 (5.45)	142–175	10.5 (1.98)	10.1–11.0
Drug–drug interaction effects (% decrease)						
chloroquine						
*V*/*F*_PRQ_	13.8 (15.8)	9.62–17.9	NA	NA	NA	NA
dihydroartemisinin/piperaquine						
*V*/*F*_PRQ_	15.0 (21.9)	8.34–20.8	16.0 (18.2)	10.8–22.1	22.3 (13.6)	15.8–27.6
pyronaridine/artesunate						
CL/*F*_PRQ_	18.0 (19.1)	10.5–23.7	19.5 (19.6)	11.6–26.5	NA	NA
*V*/*F*_PRQ_	24.3 (18.0)	14.8–31.8	27.5 (15.7)	18.4–35.1	10.2 (25.2)	4.79–14.8
Random effects [%CV (%RSE)]						
IIV *F*	15.8 (11.3)	12.0–18.9	26.7 (14.3)	18.5–34.0	13.8 (11.2)	10.4–16.4
IIV CL/*F*_PRQ_	16.0 (16.3)	11.0–21.5	NA	NA	NA	NA
IIV *V*/*F*_PRQ_	15.2 (24.9)	7.52–22.5	19.0 (13.4)	13.3–23.2	9.90 (17.4)	6.26–12.6
IIV CL/*F*_CPRQ_	19.1 (9.99)	15.2–22.6	25.9 (15.1)	17.8–33.4	24.5 (13.7)	17.8–31.0
IOV MTT	51.8 (6.05)	44.9–58.5	44.1 (6.95)	37.2–50.2	48.3 (6.47)	41.1–54.6
IOV *F*_m_	39.6 (8.37)	31.8–46.1	53.4 (11.2)	40.4–65.9	41.7 (10.6)	32.1–50.8
RUV_PRQ_	42.6 (6.17)	37.0–48.4	26.5 (8.20)	22.3–31.0	37.4 (7.26)	32.1–43.5
RUV_CPRQ_	21.4 (8.33)	18.1–25.2	17.5 (11.1)	13.7–21.4	16.8 (7.34)	14.3–19.3
RUV_PRQ,CPRQ_	27.2 (8.83)	22.1–31.6	14.1 (11.8)	10.7–17.1	20.6 (11.8)	15.9–25.3

RUV, additive residual variability; NA, data not available.

a Population mean, IIV and IOV were computed from NONMEM. IIV and IOV are presented as [exp^(estimate)^  − 1]^1/2^ ^ ^× 100.

b RSE and 95% CI were computed using the non-parametric bootstrap method of the final pharmacokinetic models (*n *=* *1000).

All secondary parameters of primaquine and carboxyprimaquine calculated from final population pharmacokinetic models are presented in Table 2. Interactions with all three tested antimalarial treatments resulted in a significantly higher *C*_max_ and a significantly shorter *t*_1/2_ of racemic primaquine. A significant increase in AUC of racemic primaquine and a decrease in AUC_CPRQ_/AUC_PRQ_ were observed when primaquine was co-administered with pyronaridine/artesunate, but not with the other antimalarials.

**Table 2.** Secondary parameters of primaquine and carboxyprimaquine from the final population pharmacokinetic models

Secondary parameters, median (range)	Primaquine alone	Primaquine co-administered with antimalarial drugs		
		chloroquine	dihydroartemisinin/ piperaquine	pyronaridine/artesunate
Racemic				
*C*_max PRQ_ (ng/mL)	113 (48.7–219)	120 (71.7–197)**	131 (56.4–219)**	144 (94.5–292)**
*T*_max PRQ_ (h)	1.96 (0.721–3.67)	1.73 (0.745–3.33)**	1.31 (0.784–2.97)	1.35 (0.744–2.51)
*t*_1/2 PRQ_ (h)	6.66 (4.51–9.02)	6.11 (4.60–7.77)**	5.88 (4.51–7.10)**	5.74 (4.16–7.20)**
AUC_0–48 PRQ_ (ng·h/mL)	1190 (629–2680)	1170 (745–2300)	1160 (506–2280)	1370 (648–2900)*
AUC_0–∞ PRQ_ (ng·h/mL)	1200 (638–2730)	1180 (750–2340)	1170 (510–2300)	1380 (648–2910)*
*C*_max CPRQ_ (ng/mL)	1040 (725–1550)	1070 (865–1560)**	1060 (798–1440)**	1220 (781–1630)**
*T*_max CPRQ_ (h)	10.5 (5.62–15.7)	9.84 (7.54–14.6)*	9.83 (5.62–15.0)	8.87 (7.06–11.2)**
*t*_1/2 CPRQ_ (h)	15.6 (10.2–23.2)	16.6 (10.2–20.8)	15.3 (12.6–23.2)	13.8 (12.2–19.2)
AUC_0–48 CPRQ_ (ng·h/mL)	35 800 (21 600–55 400)	33 500 (24 500–49 200)**	33 600 (22 000–50 400)*	34 600 (20 600–53 800)**
AUC_0–∞ CPRQ_ (ng·h/mL)	43 700 (24 500–73 100)	45 400 (28 500–63 600)**	41 700 (24 900–64 500)**	45 700 (24 500–73 300)**
AUC_CPRQ_/AUC_PRQ_	35.2 (13.7–66.2)	35.9 (14.4–56.5)	36.8 (24.4–64.2)	32.9 (16.0–41.8)*
(+)-*S*-enantiomer				
*C*_max PRQ_ (ng/mL)	77.9 (36.1–145)	NA	89.5 (36.1–148)**	106 (60.1–198)**
*T*_max PRQ_ (h)	1.52 (0.869–3.00)	NA	1.37 (0.816–2.77)	1.35 (0.718–2.53)
*t*_1/2 PRQ_ (h)	7.07 (4.53–9.33)	NA	6.21 (4.87–7.84)**	6.03 (4.08–8.38)**
AUC_0–48 PRQ_ (ng·h/mL)	870 (385–1960)	NA	886 (372–1810)	1000 (402–2110)**
AUC_0–∞ PRQ_ (ng·h/mL)	878 (386–2020)	NA	890 (372–1840)	1010 (403–2130)**
*C*_max CPRQ_ (ng/mL)	51.2 (28.1–86.6)	NA	55.8 (39.9–93.6)**	52.7 (30.4–84.0)**
*T*_max CPRQ_ (h)	10.7 (5.21–18.7)	NA	9.59 (5.21–17.7)**	10.0 (5.79–13.0)**
*t*_1/2 CPRQ_ (h)	13.0 (9.31–25.5)	NA	12.8 (8.78–25.5)	13.0 (9.31–22.0)
AUC_0–48 CPRQ_ (ng·h/mL)	1660 (753–3120)	NA	1710 (1030–3240)**	1690 (760–3130)**
AUC_0–∞ CPRQ_ (ng·h/mL)	1980 (816–4340)	NA	1990 (1080–4290)**	1990 (816–4360)**
AUC_CPRQ_/AUC_PRQ_	1.98 (1.35–4.40)	NA	2.16 (1.31–4.44)	1.86 (1.21–3.14)**
(−)-*R*-enantiomer				
*C*_max PRQ_ (ng/mL)	46.1 (22.6–77.5)	NA	54.1 (22.6–77.5)**	57.2 (43.3–101)*
*T*_max PRQ_ (h)	1.49 (0.851–3.13)	NA	1.25 (0.762–2.87)	1.41 (1.05–2.17)
*t*_1/2 PRQ_ (h)	4.66 (3.81–5.81)	NA	3.84 (3.36–4.56)**	4.26 (3.81–5.07)
AUC_0–48 PRQ_ (ng·h/mL)	349 (193–600)	NA	328 (151–481)	383 (246–660)
AUC_0–∞ PRQ_ (ng·h/mL)	350 (193–600)	NA	328 (151–481)	384 (246–660)
*C*_max CPRQ_ (ng/mL)	989 (671–1460)	NA	983 (771–1320)**	1120 (719–1480)**
*T*_max CPRQ_ (h)	8.70 (5.69–13.9)	NA	8.23 (5.16–12.5)*	8.19 (6.77–10.1)**
*t*_1/2 CPRQ_ (h)	18.0 (14.5–37.9)	NA	17.9 (14.5–37.9)	17.2 (14.8–25.3)
AUC_0–48 CPRQ_ (ng·h/mL)	33 100 (19 800–51 400)	NA	30 800 (20 000–46 100)**	33 600 (20 400–52 200)
AUC_0–∞ CPRQ_ (ng·h/mL)	42 100 (22 800–74 200)	NA	40 900 (22 800–66 600)**	45 000 (23 400–74 200)
AUC_CPRQ_/AUC_PRQ_	109 (72.2–200)	NA	130 (74.4–254)	113 (68.7–180)

Secondary parameter estimates were calculated from the individual empirical Bayes *post hoc* estimates of the primary pharmacokinetic parameters.

AUC_CPRQ_/AUC_PRQ_, metabolite/parent drug AUC ratio; NA, data not available.

* *P *<* *0.05

** *P *<* *0.01 by Wilcoxon signed rank test.

Co-administration with dihydroartemisinin/piperaquine and pyronaridine/artesunate resulted in a significantly higher *C*_max_ of both (+)-*S*-primaquine and (−)-*R*-primaquine, and a significantly shorter *t*_1/2_ of (+)-*S*-primaquine. Only co-administration of dihydroartemisinin/piperaquine resulted in a significantly shorter *t*_1/2_ of (−)-*R*-primaquine. The AUC of (+)-*S*-primaquine was increased significantly by co-administration with pyronaridine/artesunate, which had led to a decrease in AUC_CPRQ_/AUC_PRQ_.

**Figure 2.**
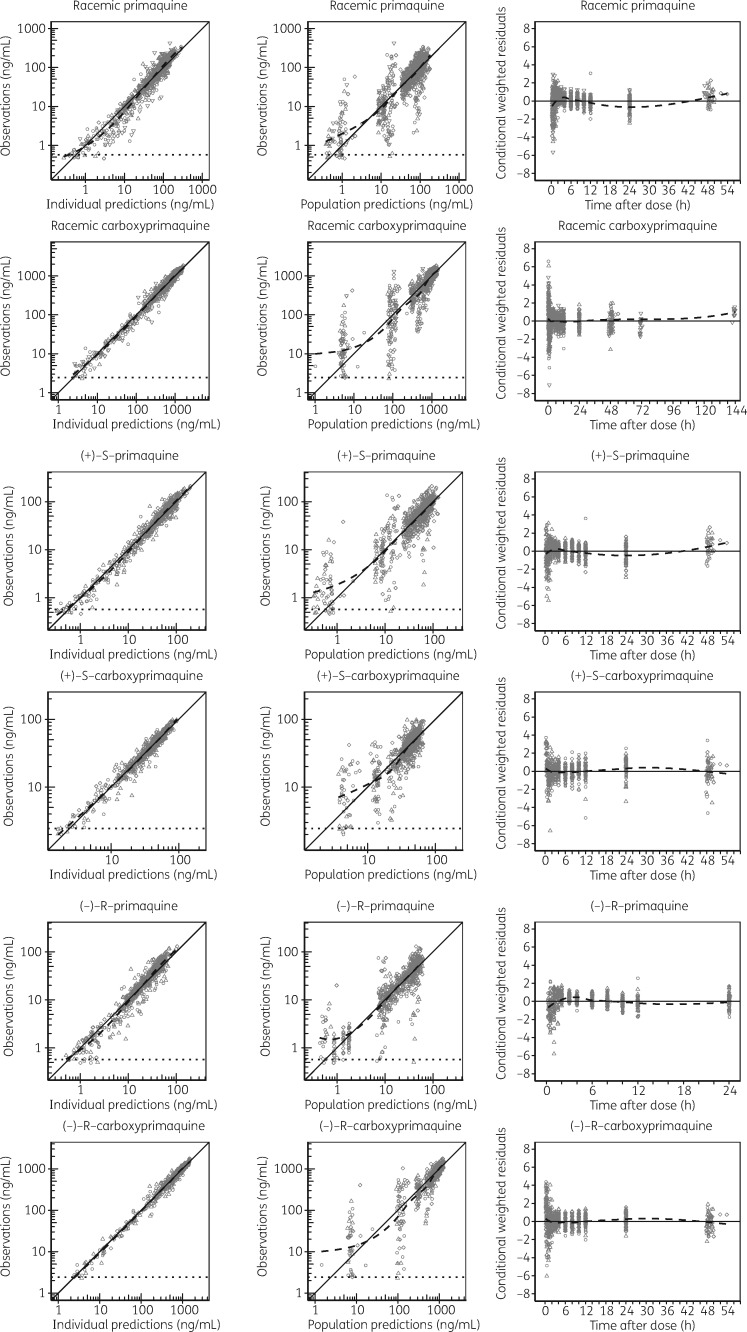
GOF diagnostics of the final population pharmacokinetic models of racemic and enantiomeric primaquine and carboxyprimaquine. The observed concentrations, population predictions and individual predictions are presented on a logarithmic (base 10) scale. Open circles, observed data for primaquine alone; downward triangles, observed data for primaquine with chloroquine; upward triangles, observed data for primaquine with dihydroartemisinin/piperaquine; diamonds, observed data for primaquine with pyronaridine/artesunate; broken lines, locally weighted least-squares regressions; solid lines, lines of identity; dotted horizontal lines, LLOQ.

**Figure 3.**
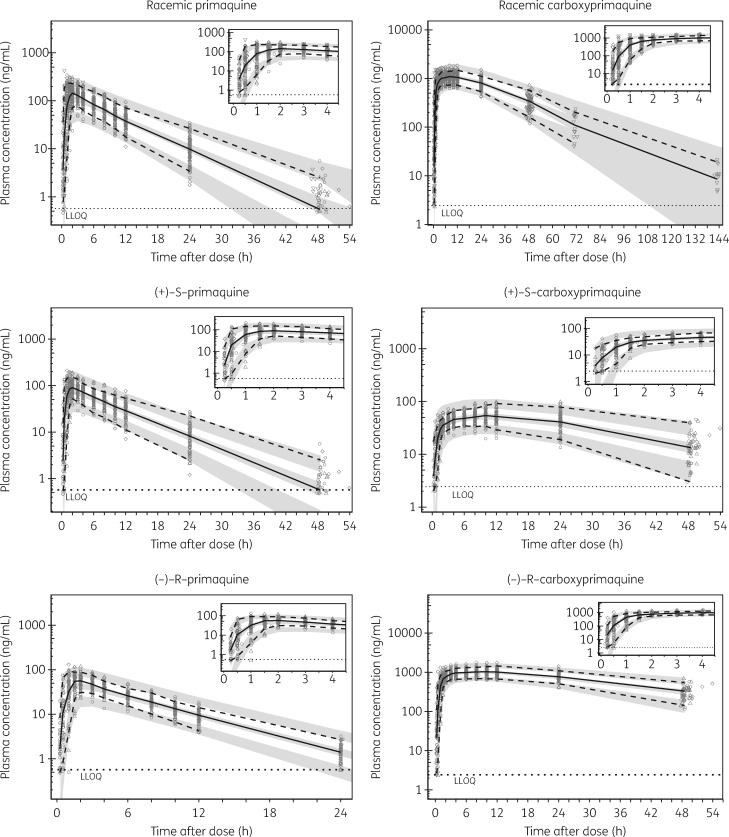
VPCs of the final population pharmacokinetic models describing racemic and enantiomeric primaquine and carboxyprimaquine. Open circles, observed data for primaquine alone; downward triangles, observed data for primaquine with chloroquine; upward triangles, observed data for primaquine with dihydroartemisinin/piperaquine; diamonds, observed data for primaquine with pyronaridine/artesunate; solid lines, 50th percentiles of the observed data; broken lines, 5th and 95th percentiles of the observed data; shaded areas, 95% CIs of simulated (*n *=* *2000) 5th, 50th and 95th percentiles; dotted horizontal lines, LLOQ. Concentrations are displayed on a logarithmic (base 10) scale.

In all three models, IIV and IOV estimates <10% were fixed to zero variability and omitting them did not affect the overall model fits. The final pharmacokinetic model parameters were estimated with good precision (Table 1). Relatively low η shrinkages (<15%) were observed for all of the included random effects except IIV on *V*/*F*_PRQ_ (24.6%) in the racemic model. The ε shrinkages were generally low (<10%) for both primaquine and carboxyprimaquine in racemic and enantiomeric models. GOF plots showed no major model misspecification (Figure 2). VPCs of the final population models are shown in Figure 3. The final population parameter estimates with 95% CIs obtained from the bootstrap analysis are shown in Table 1.

### Drug–drug interactions

**Figure 4.**
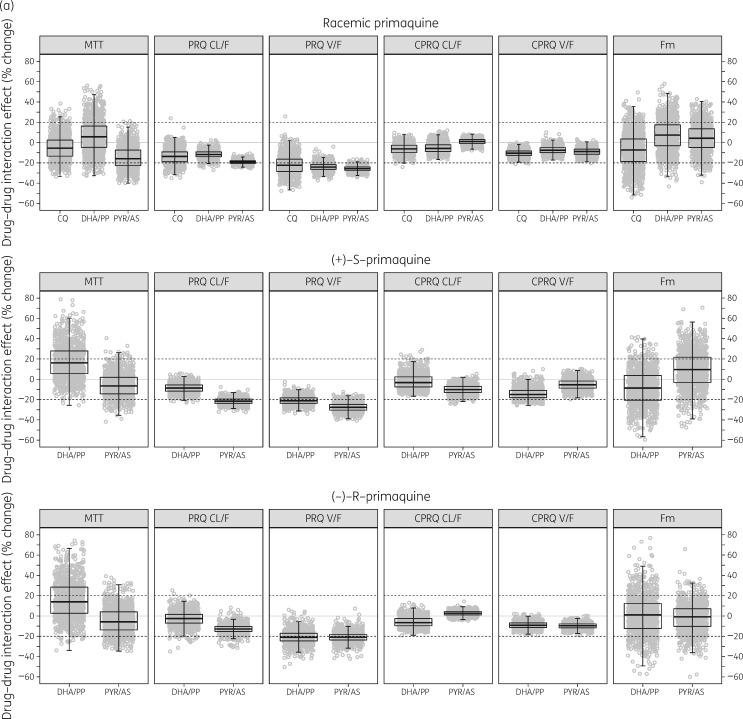

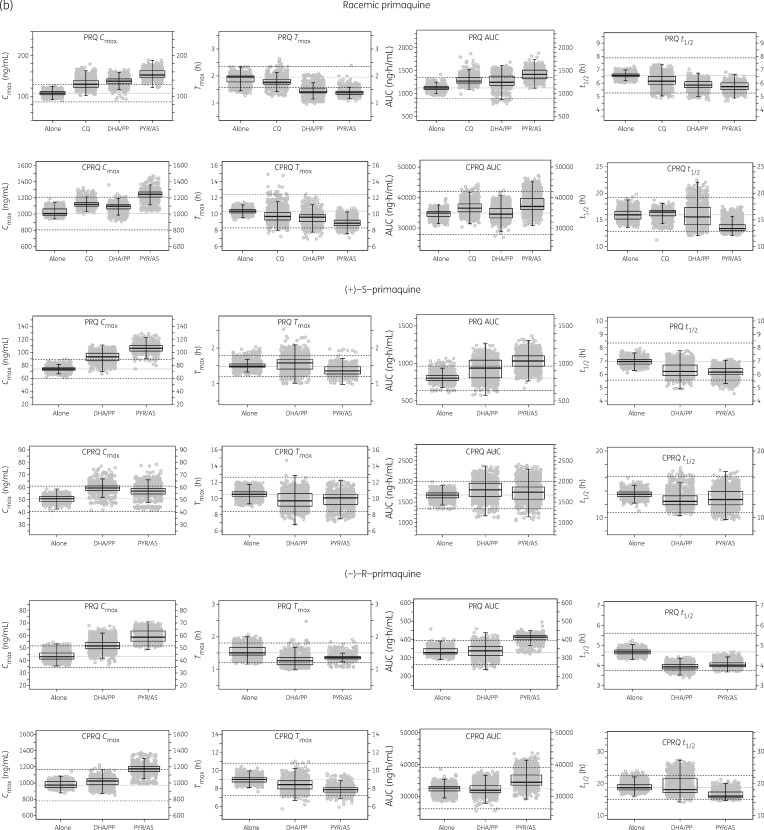
Effect of co-administration of chloroquine (CQ), dihydroartemisinin/piperaquine (DHA/PP) or pyronaridine/artesunate (PYR/AS) when evaluated on (a) primary and (b) secondary pharmacokinetic parameters of primaquine and carboxyprimaquine using a full covariate approach. The dashed lines represent ±20% differences in parameter estimates caused by co-administration of antimalarial drugs.

Co-administration of primaquine with other antimalarial treatments was found to affect the pharmacokinetic properties of primaquine significantly, but it did not alter the pharmacokinetics of carboxyprimaquine. The effects of each blood-stage antimalarial co-administration were evaluated further using a full covariate approach and bootstrap diagnostics, as shown in Figure 4(a and b).

The *V*/*F* of racemic primaquine was decreased by a median (95% CI) of 22.0% (2.24%–39.9%), 24.0% (15.0%–31.5%) and 25.7% (20.3%–31.1%) in the presence of chloroquine, dihydroartemisinin/piperaquine and pyronaridine/artesunate, respectively. The CL/*F* of racemic primaquine decreased by a median of 19.1% (14.5%–22.8%) in the presence of pyronaridine/artesunate. This resulted in a significant increase in *C*_max_ and AUC of primaquine and carboxyprimaquine.

The *V*/*F* of (+)-*S*-primaquine was decreased by a median of 21.0% (12.0%–28.8%) and 27.6% (18.2%–35.9%) in the presence of dihydroartemisinin/piperaquine and pyronaridine/artesunate, respectively. The CL/*F* of (+)-*S*-primaquine was decreased by a median of 21.5% (14.9%–27.5%) in the presence of pyronaridine/artesunate.

The *V*/*F* of (−)-*R*-primaquine was decreased by a median of 20.8% (5.93%–34.8%) and 20.9% (9.61%–31.8%) in the presence of dihydroartemisinin/piperaquine and pyronaridine/artesunate, respectively. However the CL/*F* of (−)-*R*-primaquine was not significantly affected by the presence of dihydroartemisinin/piperaquine or pyronaridine/artesunate. It was not possible to evaluate the enantiospecific impact of chloroquine since only racemic concentrations were measured in this clinical study. 

## Discussion

This study described successfully the pharmacokinetic properties of racemic and enantiomeric primaquine and its major metabolite, carboxyprimaquine, after oral administration of primaquine racemate using a population pharmacokinetic modelling approach. We confirmed that the pharmacokinetics of primaquine, but not carboxyprimaquine, were altered when it was co-administered with chloroquine, dihydroartemisinin/piperaquine or pyronaridine/artesunate to healthy volunteers.

The structural model, comprising a series of transit absorption compartments followed by one disposition compartment for primaquine and one disposition compartment for carboxyprimaquine, was able to characterize adequately the plasma concentration profiles of a racemic mixture and both (+)-*S*- and (−)-*R*-enantiomers. Including pre-systemic metabolism of primaquine in the model improved the model fit significantly, consistent with a recent published pharmacokinetic study.^35^

Clearance and volume parameters were allometrically scaled by body weight, which has also been described in previously published pharmacokinetic models of primaquine.^32^^,^^35^

The data presented here show that the pharmacokinetic properties of primaquine are enantiospecific. (−)-*R*-primaquine was found to have relatively higher CL/*F* and *V*/*F* than (+)-*S*-primaquine, resulting in a 2-fold higher exposure to (+)-*S*-primaquine, consistent with previous reports.^21^^,^^36^ However, there were substantially different exposures to the carboxyprimaquine enantiomers, resulting in a 1000-fold higher exposure to (−)-*R*-carboxyprimaquine compared with (+)-*S*-carboxyprimaquine. Considering that MAO-A, based on *in vitro* recombinant enzyme assays, is responsible for the formation of carboxyprimaquine, this observation may suggest that (+)-*S*-primaquine would be a more important substrate than (−)-*R*-primaquine for formation of an active metabolite through the CYP2D6-mediated metabolism pathway (assuming approximately similar clearance and volume values for enantiomeric carboxyprimaquine). Chloroquine, dihydroartemisinin and pyronaridine have all been reported to inhibit CYP2D6 activity, though with different degrees of inhibition capacity.^37–39^ Consistent with these reports, we found that co-administration of chloroquine, dihydroartemisinin/piperaquine and pyronaridine/artesunate caused a small reduction in overall clearance, with pyronaridine/artesunate showing the largest inhibition (19.3%). The interaction effect was more pronounced on the metabolism of (+)-*S*-primaquine compared with that of the (−)-*R*-enantiomer. This finding is consistent with the earlier report of enantiomeric preference in the metabolism of primaquine.^20^

In addition, co-administration of these drugs also caused a decrease in *V*/*F*_PRQ._ This effect was more prominent with (+)-*S*-primaquine compared with (−)-*R*-primaquine. Possible explanations include tissue binding site competition or inhibition of transporter-mediated uptake into cellular compartments impeding tissue accumulation of primaquine. Although this interaction might cause reduced primaquine transport to infected hepatocytes and subsequently result in the decrease in active metabolite exposure by preventing metabolism, potentiation of the anti-relapse efficacy of primaquine by chloroquine was reported by Alving *et al.*^40^ Since primaquine is a substrate of the efflux transporter P-glycoprotein (P-gp), inhibition of P-gp on the surface of hepatocytes by co-administered drugs would be expected to retain primaquine in the liver.^41^

On the other hand, inhibition of the P-gp transporter in the luminal membrane of the small intestine might also promote primaquine absorption into enterocytes, which could explain the reduced MTT of primaquine when co-administered with the P-gp inhibitor pyronaridine. However, this increase in absorption was minimal as primaquine is normally well absorbed.^7^

Accurate prediction of potential drug–drug interactions of primaquine is challenging as it has been shown to interact with other commonly used antimalarial drugs through several drug transporters and the CYP2D6 enzyme pathway, all of which are potentially affected by genetic polymorphisms and therefore increase IIV pharmacokinetics. This suggests that the efficacy and safety of primaquine-containing combination therapies should be assessed further in individuals with these polymorphisms.

The first limitation of the study is that the active metabolite(s) of primaquine, responsible for its main pharmacodynamic effects, is unknown and there is still not a clear understanding of the enantioselective metabolism of primaquine. This limits the ability of pharmacokinetic models to predict antiparasitic efficacy and haematological toxicity due to drug–drug interactions. Second, it was not possible to evaluate the enantiospecific interaction effect mediated by co-administration with chloroquine since the enantiomeric plasma concentrations were not available. However, a relatively higher inhibitory effect of chloroquine on (+)-*S*-primaquine than on the (−)-*R*-enantiomer might be expected based on observations from previous *in vitro* studies using recombinant human CYP2D6 and MAO incubations.^42^ Chloroquine inhibited (+)-*S*-primaquine metabolism to form CYP2D6-dependent metabolites but it did not affect MAO-mediated metabolism. Lastly, this study was performed in healthy volunteers, who may not be representative for assessing the effects of acute drug–drug interactions in patients. Despite this, a previous study confirmed that co-administration of primaquine (30 mg of base daily for 14 days) with either dihydroartemisinin/piperaquine (standard daily dosing of 120 mg of dihydroartemisinin and 960 mg of piperaquine phosphate for 3 days) or pyronaridine/artesunate (single daily dosing of 540 mg of pyronaridine phosphate and 180 mg of artesunate for 3 days) did not compromise primaquine safety or efficacy for radical cure of vivax malaria in patients.^43^

### Conclusions

We have successfully characterized the pharmacokinetic properties of racemic and enantiomeric primaquine and carboxyprimaquine using population pharmacokinetic modelling. The pharmacokinetic properties of the enantiomers showed substantial differences in the exposure to primaquine and carboxyprimaquine enantiomers. All evaluated antimalarial drugs showed a drug–drug interaction with primaquine, resulting in an ∼10%–30% increase in exposure to primaquine. This pharmacokinetic interaction was also enantiospecific. The developed pharmacokinetic models presented here can be used for further assessment of efficacy and safety of primaquine and other antimalarial drug combination therapies in future studies.
